# Tacrolimus Inhibits NF-κB Activation in Peripheral Human T Cells

**DOI:** 10.1371/journal.pone.0060784

**Published:** 2013-04-01

**Authors:** Ramin Vafadari, Rens Kraaijeveld, Willem Weimar, Carla C. Baan

**Affiliations:** Department of Internal Medicine, Erasmus MC, University Medical Center Rotterdam, Rotterdam, The Netherlands; Rush University, United States Of America

## Abstract

The calcineurin inhibitor, tacrolimus (TAC), inhibits the protein phosphatase activity of calcineurin, leading to suppression of the nuclear translocation of NFAT and inhibition of T cell activation. Apart from NFAT also the transcription factor NF-κB plays a key functional role in T cell activation. Therefore, blockade of the NF-κB activation cascade by immunosuppressive drugs prevents immune activation. Here we studied whether TAC blocks NF-κB activation in peripheral human T cells. After anti-CD3/CD28-activation of T cells from healthy volunteers, NF-κB (p65) phosphorylation was measured by flow cytometry in CD3+ T cells, CD4+ helper T cells and CD8+ cytotoxic T cells in the absence and presence of TAC 10 ng/mL, sotrastaurin 500 nM (positive control) and mycophenolic acid 10 µg/mL (negative control; n = 6). NF-κB transcriptional activity was measured by ELISA and intracellular TNFα protein, a downstream target, was measured by flow cytometry to assess the functional consequences of NF-κB blockade. Anti-CD3/28-activation induced NF-κB phosphorylation in CD3+ T cells, CD4+ T cells and CD8+ T cells by 34% (mean), 38% and 30% resp. (p<0.01). Sotrastaurin inhibited NF-κB activation in the respective T cell subsets by 93%, 95% and 86% (p<0.01 vs. no drug), while mycophenolic acid did not affect this activation pathway. Surprisingly, TAC also inhibited NF-κB phosphorylation, by 55% (p<0.01) in CD3+ T cells, by 56% (p<0.01) in CD4+ T cells and by 51% in CD8+ T cells (p<0.01). In addition, TAC suppressed NF-κB DNA binding capacity by 55% (p<0.05) in CD3+ T cells and TNFα protein expression was inhibited in CD3+ T cells, CD4+ T cells and CD8+ T cells by 76%, 71% and 93% resp. (p<0.01 vs. no drug), confirming impaired NF-κB signaling. This study shows the suppressive effect of TAC on NF-κB signaling in peripheral human T cell subsets, measured at three specific positions in the NF-κB activation cascade.

## Introduction

Tacrolimus (TAC) is a commonly used drug in the immunosuppressive regimen following solid organ transplantation [1]. It is highly effective in the prevention of graft rejection [2], [3], [4], [5], an immunological process where T cells are often involved [6]. In these cells TAC binds to and inhibits calcineurin after forming a complex with the immunophilin FKBP12 (FK506 binding protein) [7]. Calcineurin is activated after engagement of the T cell receptor (TCR) and co-stimulation (i.e. CD28-receptor stimulation). Activation of these receptors leads to depletion of endoplasmatic reticulum calcium-stores into the cytosolar cell compartment and following calcium influx via CRAC (calcium release activated calcium current) channels to slowly replenish the calcium levels in the endoplasmic reticulum [8]. Activation of the calcium-dependent calcineurin leads to dephosphorylation and translocation of nuclear factor of activated T cells (NFAT) into the nucleus. NFAT binds in conjunction with AP-1 to DNA binding sites and initiates transcription of pro-inflammatory cytokines (e.g. IL-2 and IFNγ) [8].

Since its introduction over two decades ago, textbooks without exception have attributed the immunosuppressive effect of TAC to inhibition of the calcineurin/NFAT pathway. Interestingly some studies have linked inhibition of the calcineurin pathway to suppression of NF-κB signaling in the Jurkat T cell line [9], [10], [11]. The interaction between these two major activation pathways is still under investigation [12] and suggests that TAC also suppresses T cell activation via the NF-κB pathway. In resting T cells IκB (inhibitor of κB) proteins keep NF-κB inactive by masking its nuclear localization sequence. After TCR-engagement and co-stimulation, the inhibitory IκB proteins are degraded by IKK (IκB kinase) complexes via the 26S proteasome. This enables the release of NF-κB hetero-dimers primarily consisting of NF-κB1 (p50) and RelA (p65) to enter the nucleus and bind to the κB sites within promoters and enhancers thereby initiating transcription of adhesion molecules, chemokines, regulators of apoptosis and pro-inflammatory cytokines, e.g. tumor necrosis factor (TNF) α [13], [14]. Hence, the NF-κB signaling pathway is in-dispensable in T cell biology and plays an important role in their development, activation and survival [15], [16]. Consequently NF-κB is an important mediator of rejection processes after solid organ transplantation [17], [18].

The current study is the first to report on the effect of TAC on the NF-κB activation pathway by quantitative analysis of NF-κB phosphorylation in human primary T cell subsets. In addition, the effects of TAC on NF-κB DNA binding activity and NF-κB dependent cytokine production were analyzed. The analysis was conducted in anti-CD3/CD28 activated peripheral T cells and the effects were compared to those of sotrastaurin which inhibits protein kinase C (PKC), the enzyme that mediates NF-κB-activation after TNFα receptor engagement and TCR activation [19], [20]. The immunosuppressant mycophenolic acid (MPA) which blocks *de novo* biosynthesis of purine nucleotides and lymphocyte proliferation by suppressing the enzyme inosine monophosphate dehydrogenase, was used as negative control [21].

## Materials and Methods

### Isolation and stimulation of human peripheral T cells

The study protocol was approved by the local ethics committee of the Erasmus medical center and written informed consent was obtained from healthy volunteers. They had no clinical signs of viral or bacterial infections and did not take any medication in the 3 days up to blood withdrawal. Peripheral blood mononuclear cells (PBMC) were isolated from freshly drawn, heparinized normal human whole blood by density-gradient centrifugation over Ficoll-paque (Amersham Pharmacia Biotech, Uppsala, Sweden) and frozen at −135°C in RPMI 1640-DM (Gibco BRL, Scotland, UK) supplemented with 2 mM/L L-glutamine (Gibco BRL), 100 IU/mL penicillin (Gibco BRL), 100 ug/mL streptomycin (Gibco BRL), 10% pooled human serum (Blood Bank, Rotterdam, the Netherlands) and 10% dimethylsulfoxide (Merck, Schuchardt, Germany). Defrosted PBMC were washed twice with RPMI 1640 (Gibco BRL, Scotland, UK), supplemented with 100IU/mL penicillin (Gibco BRL) and 100 ug/mL streptomycin (Gibco BRL). Purified T cells were isolated by negative selection using MACS magnetic cell separation with a pan T cell isolation kit II (Miltenyi Biotech, Auburn, CA) according to manufacturer’s instructions. Negative selection was used to avoid possible T cell activation by binding to beads. Purity of the isolated T cells was determined by flow cytometry using a CD3-AmCyan monoclonal antibody (mAb; BD Biosciences, San Jose, CA), on a FACS Canto II flow cytometer (BD Biosciences) along with facs Diva, version 6.0 software (BD Biosciences). For each experiment the purity of the magnetically isolated CD3+ T cells was at least 98%. Cells were rested overnight in IMDM medium supplemented with 0.5 nM β-mercaptopurin (Sigma-Aldrich, Steinheim, Germany) and 10% heat inactivated fetal bovine serum (FBS) (BioWhittaker, Verviers, Belgium).

### Phosphospecific flow cytometry

MACS isolated T cells (10^6^) were spiked for three hours at 37°C with drugs at the following final concentrations: TAC 0, 5, 10 and 50 ng/mL (Astellas Pharma Inc., Tokyo, Japan), sotrastaurin 50, 100 and 500 nM (Novartis Pharmaceuticals, Basel, Switzerland) and mycophenolic acid 10 µg/mL (Sigma-Aldrich). Cells were activated for ten minutes with mouse anti-human CD3 (UCHT1 clone, BD Pharmingen, San Diego, CA) and mouse anti-human CD28 (28.2 clone, BD Pharmingen) 1 µg each, according to manufacturer’s instructions at 37°C. Before activation the anti-human CD3 and CD28 were cross-linked on ice using a polyclonal goat anti-mouse immunoglobulin (BD Pharmingen). After activation cells were fixed for 10 min with Lyse/fix buffer (BD Biosciences). Fixed cells were permeabilized using a 70% aqueous ethanol solution and immediately cooled at −20°C. The permeabilized samples were stained in the dark with fluorochrome conjugated mAb mixtures for 30 min at room temperature. To measure the intracellular signaling pathway, we used a PE-conjugated mAb which recognizes phosphorylation at serine 529 in the activation domain of human NF-κB p65 (BD Biosciences). The p65-isoform is abundantly expressed in all mammal cells, including T cells [22], [23]. Simultaneously with intracellular staining the following mAb’s were used for cell surface staining: CD4-PB and CD8-PE-Cy7 (BD Biosciences). Samples were analyzed on a FACS Canto II flow cytometer (BD Biosciences). Ten thousand gated cell events were acquired from each tube and FMO negative control tubes were included.

### Intracellular cytokine production

MACS isolated T cells (0.5·10^6^) were spiked for one hour with: TAC 0, 10 ng/mL or sotrastaurin 500 nM. Samples were activated with 12.5 µL human T-activator CD3/CD28 Dynabeads (Invitrogen Dynal AS, Oslo, Norway) for 24 hours at 37°C. The CD3 antibody coated on the dynabeads is specific for the CD3ε-chain. Golgistop (BD Biosciences) was added during stimulation, which was stopped with EDTA, final concentration 2 µM. Beads were removed using a magnet and cells were fixed and lysed for 10 minutes with 4 mL FACS lysing solution (BD Biosciences). After washing, PBMC were permeabilized for 10 minutes with FACS perm solution II (BD Biosciences). Cells were stained and measured on the flow cytometer as described above. The following mAbs were used: CD4-PB, CD8-PE-CY7, anti-IL-2-APC (BD Biosciences) and PE conjugated anti-TNFα (BD Pharmingen).

### ELISA

Enzyme-Linked Immunosorbent Assay (ELISA) was used to analyze the effect of TAC on NF-κB DNA binding activity. MACS isolated T cells (3·10^6^) were spiked with: TAC 0, 10, 50 ng/mL ng/mL or sotrastaurin 500 nM for 1 hour. Next samples were activated for 40 minutes at 37°C with mouse anti-human CD3 (1 µg; BD Pharmingen) and mouse anti-human CD28 (1 µg; BD Pharmingen). Nuclear extracts were prepared using a nuclear extraction kit (Cayman Chemical, Ann Arbor, MN). NF-κB p65 DNA binding activity was determined by an ELISA kit (Cayman Chemical), according to the manufacturer's instructions. A bicinchoninic acid (BCA; Pierce, Rockford, IL) assay was used to determine the total protein concentration of nuclear extracts, using acetylated Bovine serum albumin (Promega, Madison, WI, USA) as standard.

### Analysis of data

Statistical analysis was performed using Graph Pad Prism 5.0 (Graph Pad Software Inc., La Jolla, CA, USA). For statistical analysis the Student’s unpaired t test was used. A value of p<0.05 was considered significant.

## Results

### NF-κB phosphorylation

**Figure 1:**
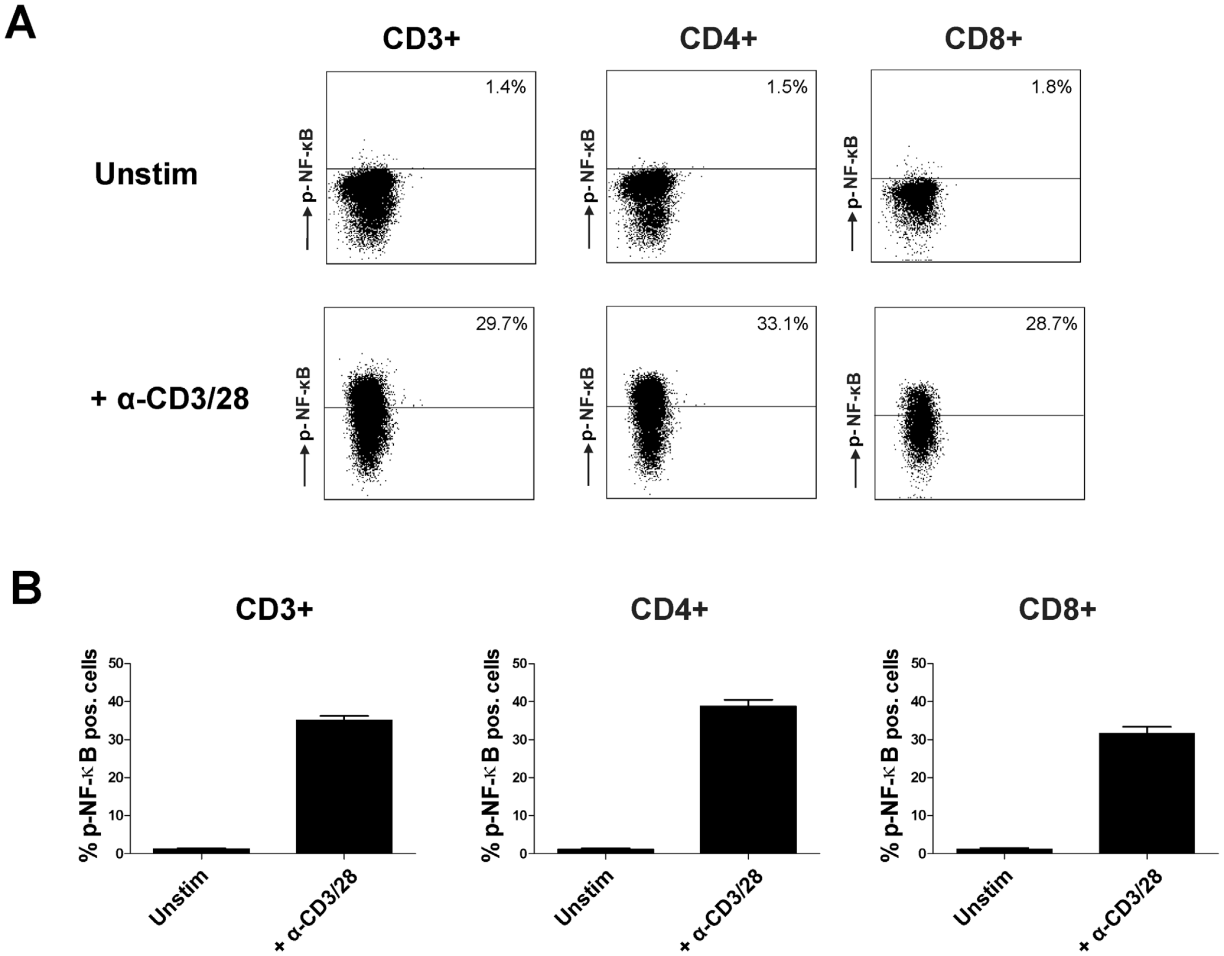
Facs analysis of NF-κB phosphorylation in T cells. The total CD3+ T cell population was acquired by MACS isolation of PBMC from healthy volunteers. **A)** Example scatter dot plots of an unstimulated sample (above) and an anti-CD3/CD28 stimulated sample to illustrate the gating strategy for analysis of percentages CD3+, CD4+ and CD8+ T cells which express phosphorylated NF-κB. Both samples were stained with a monoclonal antibody against phosphorylated NF-κB p65. **B)** The average percentages of cells with elevated NF-κB phosphorylation in unstimulated and stimulated samples without drug are depicted as mean ± SEM of 6 independent experiments.

In the first set of experiments signaling activation was studied via phoshorylation of NF-κB p65 at serine 529. The latter is only phosphorylated when the inhibitory IκB protein is released from the NF-κB dimer complex [24], [25]. NF-κB activation was measured by phosphospecific flow cytometry as the percentage of cells in which NF-κB phosphorylation was induced compared to the unstimulated sample. Figure 1A shows an example of the analysis of an unstimulated sample and an anti-CD3/CD28 stimulated sample, both stained with the monoclonal NF-κB antibody. Stimulation increased the percentage of phospho-NF-κB expressing CD3+ T cells from 1,3% in unstimulated samples to 35.0% in stimulated samples (mean, n = 6; p<0.01; figure 1B). In the CD4+ and CD8+ T cell subsets NF-κB phosphorylation was induced from 1.2% to 38.8% and from 1.2% to 31.6% respectively (p<0.01).

**Figure 2:**
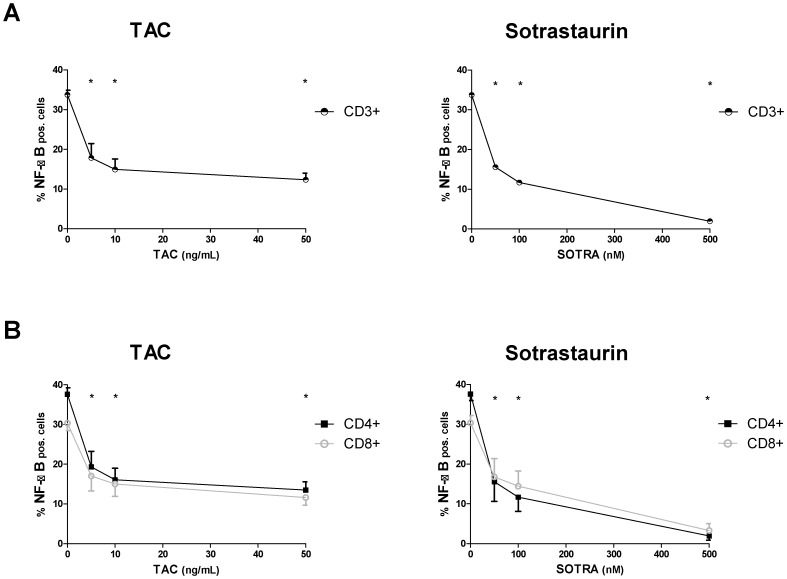
NF-κB phosphorylation inhibited at different TAC-concentrations. MACS isolated CD3+ T cells from healthy volunteers were stimulated by anti-CD3/CD28 in the presence of TAC 0, 5, 10, 50 ng/mL or sotrastaurin 50, 100 and 500 nM. Inhibition of NF-κB phosphorylation is shown for CD3+ T cells (above), CD4+ T cells and CD8+ T cells (below; mean ± SEM of 6 independent experiments).

**Figure 3:**
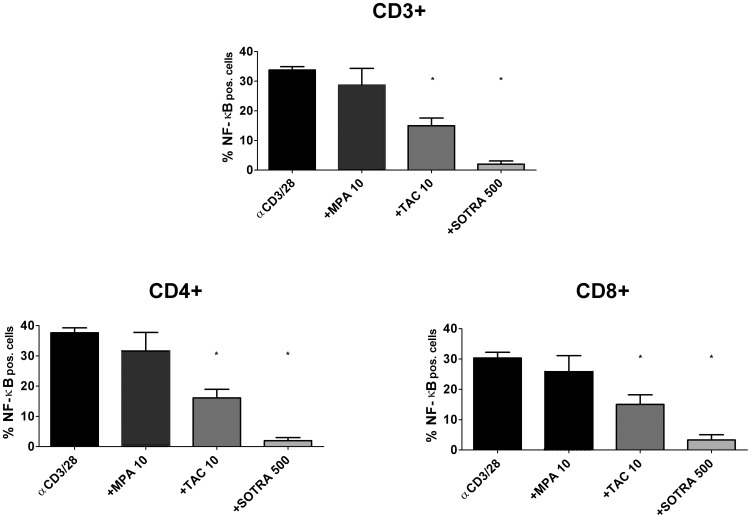
NF-κB phosphorylation inhibited by immunosuppressive drugs. The effect of the immunosuppressive drugs mycophenolic acid 10 µg/mL, TAC 10 ng/mL and sotrastaurin 500 nM on NF-κB p65 phosphorylation in peripheral T cells from healthy volunteers is depicted. Mycophenolic acid did not influence NF-κB phosphorylation (p>0.05), while the positive control sotrastaurin inhibited phosphorylation in the CD3+, CD4+ and CD8+ T cell subsets (p<0.01 compared to no drug). TAC 10 ng/mL also suppressed phosphorylation in the T cell subsets (p<0.01; mean ± SEM of 6 independent experiments).

The influence of different TAC concentrations on NF-κB phosphorylation was measured and the immunosuppressive drug inhibited NF-κB phosphorylation in a dose dependent manner in CD3+ T cells, CD4+ T cells and CD8+ T cells, as did the positive control sotrastaurin (figure 2). The negative control, mycophenolic acid, at a clinically high concentration of 10 µg/mL, did not affect NF-κB activation (p>0.05 compared to no drug; figure 3). TAC 10 ng/mL inhibited NF-κB phosphorylation by 55.0% in CD3+ T cells, by 55.7% in CD4+ T cells and by 51.2% in CD8+ T cells (p<0.01 for all T cell subsets, figure 3). The positive control, sotrastaurin 500 nM, strongly inhibited phosphorylation, by 93.9% in CD3+ T cells, by 94.0% in CD4+ T cells and by 89.7% in CD8+ T cells (p<0.01 for all T cell subsets; figure 3).

TAC also inhibited NF-κB phosphorylation in regulatory CD4+25+127− T cells (Treg; supplemental figure S1). These cells are essential for immune homeostasis by suppressing effector T cell responses [26]. NF-κB p65 is associated with induction of Foxp3 transcription [27], [28] which marks thymic Treg generation [29]. Further studies are necessary to validate whether the reduction of Treg numbers by calcineurin inhibitors [30], [31], [32] is NF-κB mediated.

### NF-κB binding to target-genes

**Figure 4:**
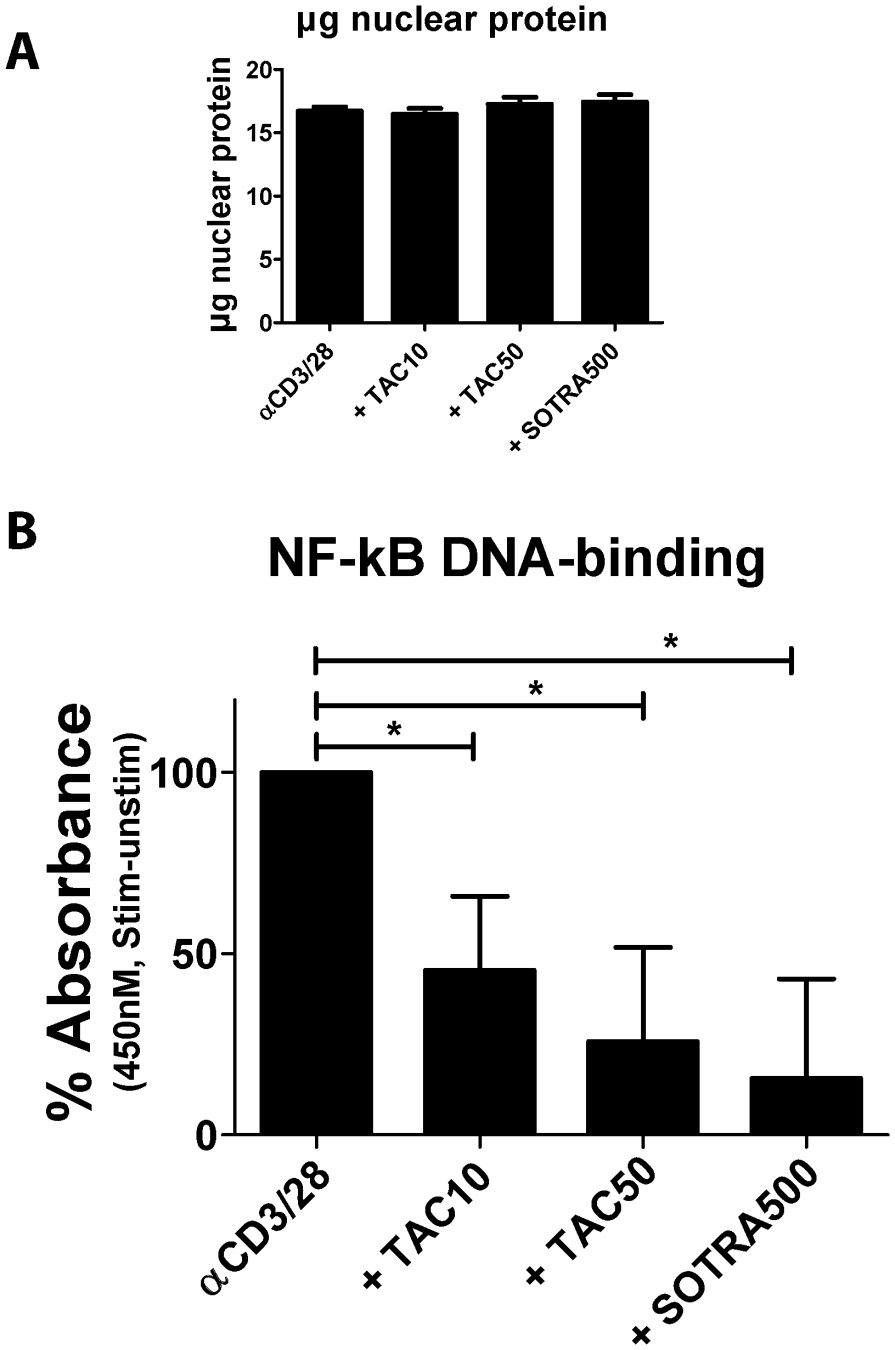
TAC inhibits NF-κB DNA-binding capacity in human T cells. MACS isolated CD3+ T cells (3.10^6^) from three healthy volunteers were stimulated in the presence of TAC 0, 10, 50 ng/mL or sotrastaurin 500 nM. **A)** The amount of nuclear protein extracted from the cells was comparable in the samples and at the different tested conditions. **B)** The DNA binding capacity of NF-κB in the nuclear fractions was inhibited by both TAC and the positive control sotrastaurin. Each bar represents the anti-CD3/CD28-stimulated NF-κB DNA binding capacity minus that of the unstimulated sample (mean ± SEM of 3 independent experiments).

After activation, phosphorylated NF-κB binds to DNA sequences called κB sites and regulates their transcription. Here we studied the downstream targets of NF-κB phosphorylation by measuring the effects of immunosuppressive drugs on the DNA binding activity of NF-κB p65 to its target-genes in CD3+ T cells. For each sample the nuclear extract of purified CD3+ T cells (3.10^6^) from healthy volunteers was prepared for analysis by ELISA. Figure 4A shows the mean amount of nuclear extract expressed in µg protein for each tested condition. This was similar at each of the tested conditions. Regarding NF-κB DNA binding activity, unstimulated samples had a mean absorbance of 1.0 and stimulated samples without drug had a mean absorbance of 2.1 (p<0.01 compared to unstimulated samples) with all individual measurements within the linear range of the assay: smaller than 3 at OD450nM. The positive control drug sotrastaurin at 500 nM inhibited the anti-CD3/28 induced DNA binding capacity of NF-κB in peripheral T cells by 84.4% (p<0.05 compared to no drug), while TAC 10 ng/mL inhibited the DNA binding capacity by 54.5% and TAC 50 ng/mL by 74.3% (both p<0.05).

### Intracellular cytokine production

**Figure 5:**
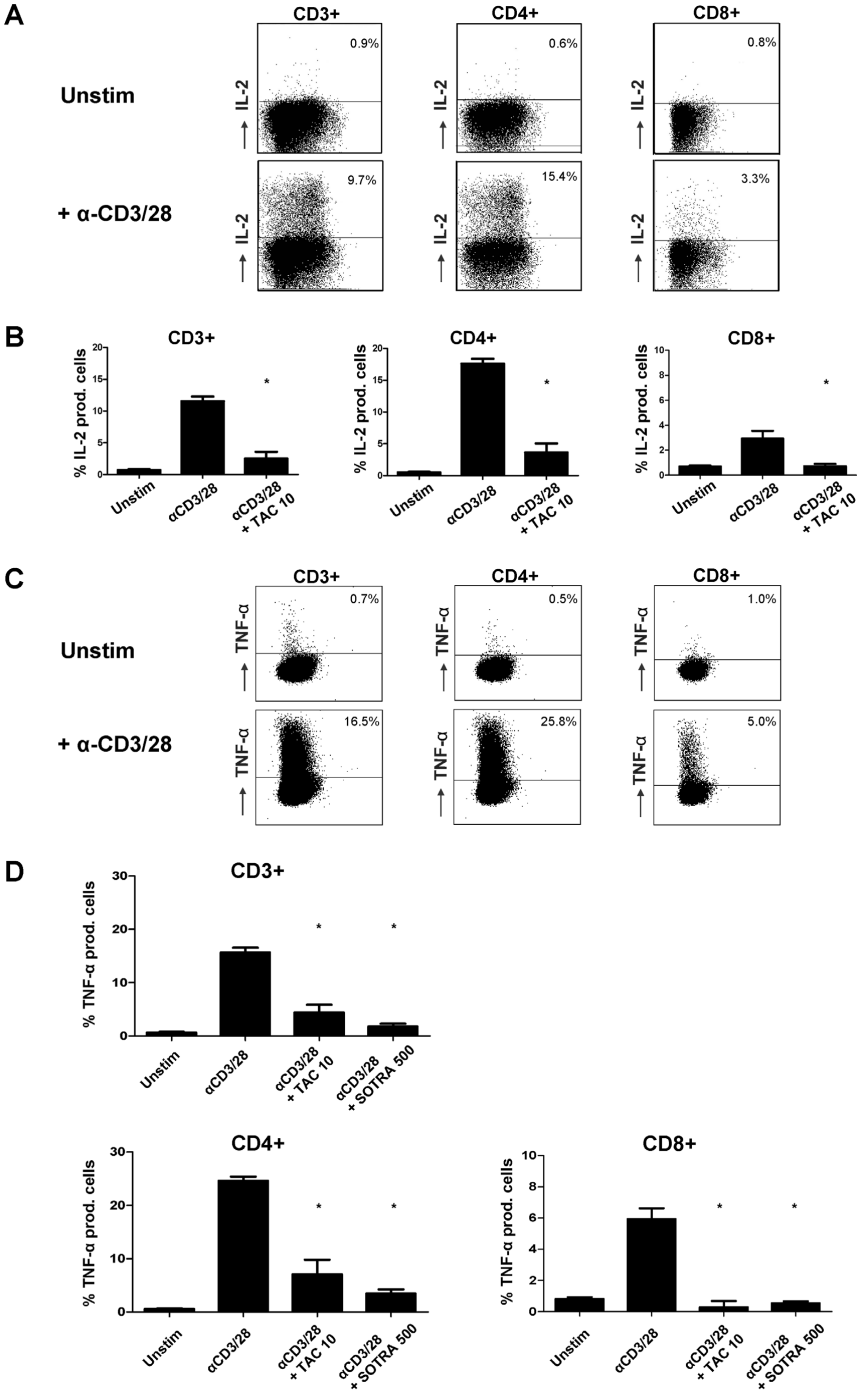
TAC induces inhibition of NF-κB dependent cytokine production. CD3+ T cells were acquired by MACS isolation of PBMC from healthy volunteers. Cells were anti-CD3/CD28 stimulated for 24 hours in the presence of TAC 0 and 10 ng/mL; and sotrastaurin 500 nM. **A)** Dot plots showing CD3+, CD4+ and CD8+ T cells with intracellular IL-2 production in an unstimulated and a stimulated sample. Both sample-types were stained for IL-2 protein expression. **B)** The percentage of IL-2 producing anti-CD3/CD28 activated T cells was fully abrogated by tacrolimus 10 ng/mL (p<0.01; mean ± SEM of 4 independent experiments). **C)** Dot plots showing CD3+, CD4+ and CD8+ T cells with intracellular TNFα production in an unstimulated and a stimulated sample. Both sample-types were stained with a monoclonal antibody against TNFα. **D)** The average percentages of TNFα producing T cells in CD3+, CD4+ and CD8+ primary T cells are depicted. TAC 10 ng/mL and sotrastaurin 500nM both inhibited the induced TNFα expression in the T cell subsets (p<0.01; mean ± SEM of 4 independent experiments).

Translocation of NF-κB into the nucleus and subsequent binding of NF-κB to its target-genes results in the downstream expression of inflammatory cytokines, chemokines and immune receptors. To examine TAC-induced effects on the expression of inflammatory cytokines, intracellular TNFα and IL-2 production was measured in T cell subsets. TNFα is an NF-κB-dependent cytokine, while IL-2 was measured as a positive control for the inhibition of the NFAT-pathway by tacrolimus. Purified CD3+ T cells were stimulated for 24 hours by the engagement of both TCR and CD28. Examples of the flow cytometric dot plots for unstimulated and stimulated samples are given in figure 5A and C. Tacrolimus abrogated the IL-2 production in CD3+, CD4+ and CD8+ T cells (p<0.01 compared to no drug, figure 5B).

For TNFα, the mean percentages of cytokine-producing T cell subsets for six experiments are depicted in figure 5D. Anti-CD3-CD28 stimulation induced TNFα production in 15.0% of CD3+ T cells, 24.0% of CD4+ T cells and 5.1% of CD8+ T cells (mean; p<0.01 unstimulated vs stimulated samples). TAC 10 ng/mL inhibited this TNFα production in CD3+, CD4+ and CD8+ T cells by 75.9%, 71.4% and 93.1% respectively (p<0.01 compared to no drug; figure 5D). Sotrastaurin inhibited the NF-κB dependent cytokine production in the T cell subsets by 88.5%, 85.7%; and 88.0% respectively (p<0.01 compared to no drug; figure 5D).

## Discussion

The transcription factor NF-κB plays an important role in T cell development, activation and survival [15], [16]. These cells are an important contributor to alloreactivity in organ transplant patients [6]. TAC suppresses T cell activation and an effect on NF-κB in T cells is eminent in unraveling its precise immunosuppressive working mechanism. Previous studies have shown that very high TAC concentrations interfere with IκB degradation [33] and IκB kinase activity in T cell lines [12], [34], without answering whether the pathway and NF-κB activation are affected in peripheral human T cells at low drug doses as used after allograft transplantation.

The current study for the first time describes the effect of clinically relevant TAC concentrations on the NF-κB signaling pathway in peripheral human T cells. We quantitatively analyzed distinct steps in the intracellular activation cascade, i.e. phosphorylation of the NF-κB molecule (figure 2) and the downstream NF-κB DNA binding activity (figure 4). Both parameters showed the inhibitory effect of TAC on NF-κB activation, including the inhibition of NF-κB phosphorylation in CD4+ helper T cells and CD8+ cytotoxic T cells. Further downstream in the activation cascade, production of the NF-κB-dependent cytokine TNFα was also suppressed by TAC in the T cell subsets (figure 5).

The traditional view on the working mechanism of TAC is that it binds a specific intracellular immunophilin FKBP12, to inhibit calcineurin phosphatase activity and subsequently the production of pro-inflammatory cytokines. Our finding that TAC also interferes with T cell activation via the NF-κB pathway can be explained by at least two theories. First, TAC can influence the NF-κB pathway via calcineurin [10], [11], [12], [35]. Biswas *et al.* [35] showed that calcineurin binds to and inactivates the cytoplasmic IκB in a mouse myoblast cell line, leading to activation of the transcription factor NF-κB and its nuclear translocation. A different mechanism of calcineurin mediated NF-κB activation was proposed by Palkowitsch *et al.* [12] who showed that downstream of TCR-CD28 engagement calcineurin controls dephosphorylation of BCL10 to facilitate formation of the Carma1, BCL10, MALT1 (CBM) complex, a pivotal step in the activation of IκB kinase. Via the CBM-complex, calcineurin phosphorylates IκB, leading to its proteosomal degradation and NF-κB activation [12], [36].

PKC phosphorylates Carma1 and is therefore also essential for the formation of the CBM complex [37]. TAC did not inhibit NF-κB activation to the same extent as the PKC inhibitor sotrastaurin (figure 3 and 4). This might be explained by partial TAC-induced calcineurin inhibition: even at 100 µg/mL this agent suppresses calcineurin activity by only 50 to 70% [38], [39]. Nevertheless, TAC-induced NF-κB suppression cannot be trivialized, since in T cells calcineurin activation is a pre-requisite for maximum IKK activity and *in vivo* IκB phosphorylation. Hence, both PKC and calcineurin are required for effective NF-κB activation [34]. Partial inhibition by TAC suggests that the affected activation pathways are not paralyzed and can still function in other essential NF-κB-dependent cell types, which is beneficial for the immunosuppressant’s efficacy and safety profile [40].

The second theory to explain how TAC influences NF-KB activation involves the MAPK (mitogen activated protein kinase) signaling pathway. Even though the precise mechanism is still under investigation, we and others have shown that TAC *in vitro* and *in vivo* inhibits the p38 MAPK activation pathway in human T cells [41], [42]. This finding can also explain NF-κB suppression by TAC as the transcription factor p38 MAPK regulates both the nuclear recruitment of NF-κB and its transcriptional activity [43], [44], [45], [46]. The signaling pathways downstream of the TCR, like the calcineurin-NFAT pathway, the PKC/NF-κB pathway and the MAPK pathway are highly interlinked and therefore it is apprehensible that TAC inhibits activation of these pathways. This is for example illustrated by studies which showed that p38 MAPK induces transcription and translation of NFAT mRNA in T cells [47] and controls NFAT transcriptional activity [48], [49].

Besides investigating the mechanism of action, the current study gives a new lead in the search for biomarkers of TAC-treatment in transplant patients. While there is a need to study intracellular activation pathways until now mostly the calcineurin-NFAT pathway has been utilized [50]. The results of our study may also influence planned clinical studies to combine sotrastaurin and TAC [51], [52]. Combining two drugs may be useful as sotrastaurin mainly inhibits the PKC/NF-κB pathway, while TAC has a strong effect on the calcineurin/NFAT pathway and as it now seems also inhibits, though to a certain extent, the NF- κB pathway. Therefore, an additional immunosuppressive effect of sotrastaurin might be expected for patients on tacrolimus maintenance therapy.

In conclusion, our study shows the suppressive effect of TAC on NF-κB signaling in peripheral human T cell subsets, measured at three specific positions in the NF-κB activation cascade. Hence, TAC is more than just a calcineurin inhibitor and a more balanced view on its immunosuppressive working mechanism is required.

## Supporting Information

Figure S1 **TAC inhibits NF-κB phosphorylation in regulatory CD4+25+127- T cells.** CD3+ T cells were acquired by MACS isolation of PBMC from healthy volunteers. **A)** Example scatter dot plots to illustrate the gating strategy for selection of CD4+CD25+127- regulatory T cells (Tregs) from the total CD4+ T cell population. B) Example scatter dot plots to illustrate the percentage of Tregs expressing phosphorylated NF-κB in an unstimulated sample and an anti-CD3/28 stimulated sample. Both stimulated and unstimulated samples were stained with a monoclonal antibody against NF-κB p65 phosphorylation. **B)** The average percentage of Tregs expressing NF-κB phosphorylation in unstimulated and stimulated samples are depicted as mean ± SEM of six independent experiments. **C)** Inhibition of phosphorylated NF-κB is shown for Tregs. TAC 10 ng/mL inhibited NF-κB phosphorylation by 44.5% in Tregs (p<0.05; mean ± SEM of 6 independent experiments).
(TIF)
