# Supplementary figures and images for: Tacrolimus Inhibits NF-κB Activation in Peripheral Human T Cells
Source: PLoS One. 2013 Apr 1;8(4):e60784. doi: 10.1371/journal.pone.0060784 (PMC3613409; doi:10.1371/journal.pone.0060784)

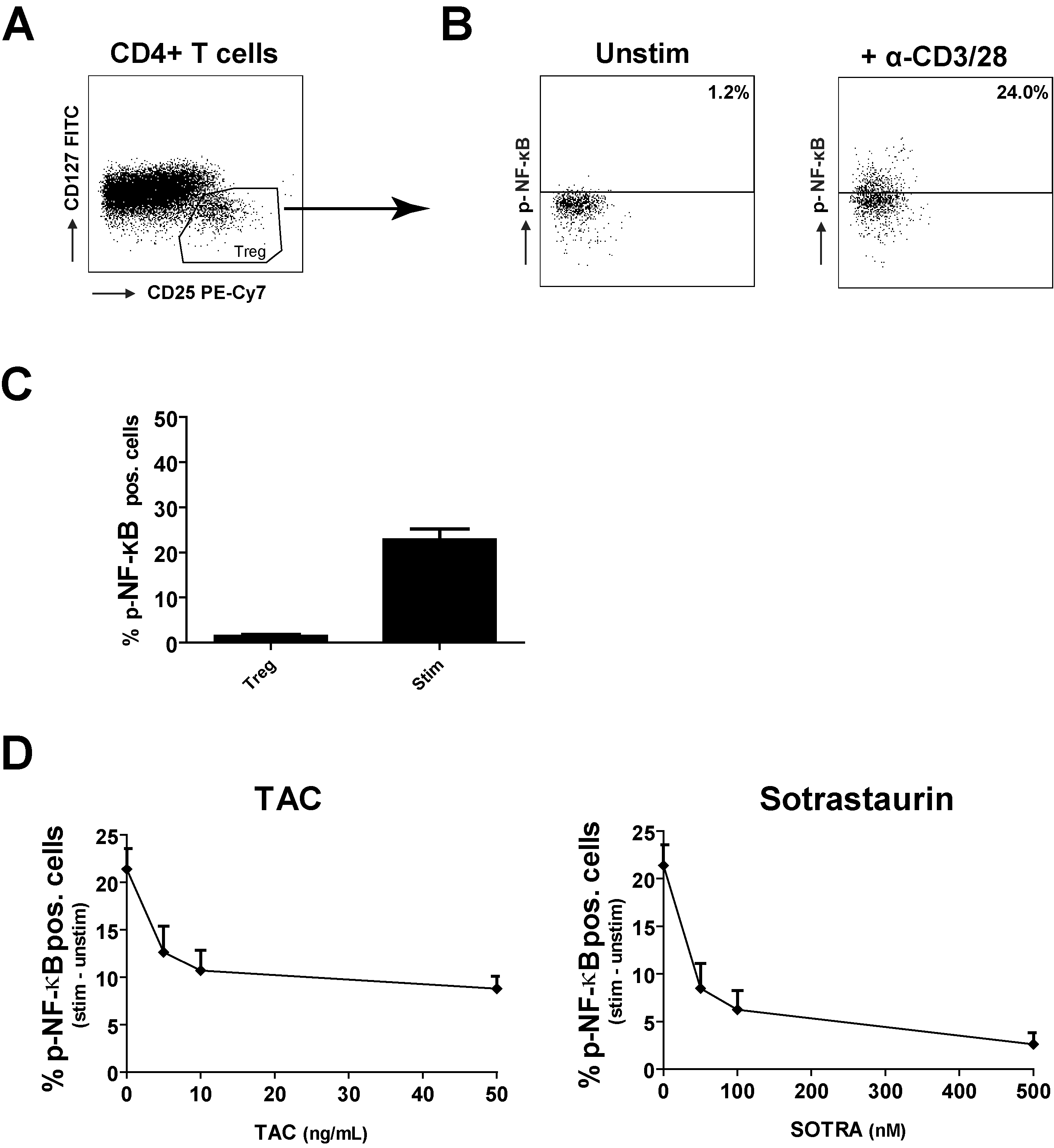

Supplement: Figure S1 — TAC inhibits NF-κB phosphorylation in regulatory CD4+25+127- T cells. CD3+ T cells were acquired by MACS isolation of PBMC from healthy volunteers. A) Example scatter dot plots to illustrate the gating strategy for selection of CD4+CD25+127- regulatory T cells (Tregs) from the total CD4+ T cell population. B) Example scatter dot plots to illustrate the percentage of Tregs expressing phosphorylated NF-κB in an unstimulated sample and an anti-CD3/28 stimulated sample. Both stimulated and unstimulated samples were stained with a monoclonal antibody against NF-κB p65 phosphorylation. B) The average percentage of Tregs expressing NF-κB phosphorylation in unstimulated and stimulated samples are depicted as mean ± SEM of six independent experiments. C) Inhibition of phosphorylated NF-κB is shown for Tregs. TAC 10 ng/mL inhibited NF-κB phosphorylation by 44.5% in Tregs (p<0.05; mean ± SEM of 6 independent experiments). (TIF) [file pone.0060784.s001.tif]
